# Enhanced immunogenicity of *Mycobacterium bovis* BCG through CRISPRi mediated depletion of AftC

**DOI:** 10.1016/j.tcsw.2022.100088

**Published:** 2022-11-11

**Authors:** Bala T.S.A. Madduri, Lauren Allen, Stephen C. Taylor, Gurdyal S. Besra, Luke J. Alderwick

**Affiliations:** a Institute of Microbiology and Infection, School of Biosciences, University of Birmingham, Edgbaston Park Road, Birmingham B15 2TT, UK; b Pathogen Immunology Group, National Infection Service, Public Health England, Porton Down, Salisbury SP4 0JG, UK; c Centre for Emerging Pathogens, Department of Medicine, Rutgers University, 225 Warren Street, Newark, NJ 07103, USA; d Charles River Laboratories, Saffron Walden, England, UK

**Keywords:** *Mycobacterium bovis* BCG, CRISPR interference, Transcriptional repression, Arabinofuranosytransferase C, Lipoarabinomannan

## Abstract

*Mycobacterium tuberculosis* causes the disease tuberculosis and affects a third of the world’s population. The recent COVID-19 pandemic exacerbated the situation with a projected 27% increase in tuberculosis related deaths. *M. tuberculosis* has an elaborate cell wall consisting of peptidoglycan, arabinogalactan and mycolic acids which shield the bacilli from the toxic bactericidal *milieu* within phagocytes. Amongst, the numerous glycosyltransferase enzymes involved in mycobacterial cell wall biosynthesis, arabinofuranosyltransferase C (*aftC*) is responsible for the branching of the arabinan domain in both arabinogalactan and lipoarabinomannan. Using Clustered Regularly Interspaced Short Palindromic Repeats interference (CRISPRi) we have generated *aftC* knockdowns in *Mycobacterium bovis* BCG and demonstrated the generation of a truncated, immunogenic lipoarabinomannan within its cell envelope. The *aftC* depleted BCG mutants were unable to form characteristic mycobacterial pellicular biofilms and elicit a potent immunostimulatory phenotype compared to wild type *M. bovis* BCG in a THP1 cell line. This study paves the way to further explore novel BCG mutants as promising vaccine boosters in preventing pulmonary tuberculosis.

## Introduction

*Mycobacterium tuberculosis* the causative agent of tuberculosis (TB), remains a global burden claiming more than a million lives each year. In 2019, 10 million new cases and 1.4 million deaths were reported by WHO. TB co-infection with HIV/AIDS and the emergence of drug resistant strains of *M. tuberculosis* has exacerbated the situation making it feasible for pulmonary viral infections, such as COVID-19 to claim millions of more lives each year (WHO report, 2020).

The *M. tuberculosis* has a robust basal cell wall structure, often referred to as the mycolyl-arabinogalactan-peptidoglycan (mAGP) complex, which sits at the core of mycobacterial cell envelope and provides a framework for the inclusion and presentation of further non-covalently bound lipids, glycolipids and lipoglycans at the surface of the cell (Alderwick et al., 2015, Ojha et al., 2008, Sambandan et al., 2013). In this regard, lipomannan (LM) and lipoarabinomannan (LAM) are two major lipoglycans presented at the periphery of the mycobacterial cell envelope, both of which play crucial roles in the pathogenesis and immunomodulation during infection (Jankute et al., 2017). The mycobacterial cell envelope and the intermediary metabolites are essential for growth, viability, and virulence of *M. tuberculosis* and hence, the enzymes involved in cell envelope synthesis are attractive drug targets (Jankute et al., 2015).

LAM is a complex phosphatidyl-*myo*-inositol based lipoglycan that contains a highly branched arabinose domain and a mannan backbone. LAM elicits potent immunomodulatory activity and contributes key molecular interactions that allows *M. tuberculosis* entry to the phagocytic compartments of macrophages through the mannose receptor and C-type lectins (Correia-Neves et al., 2019). LAM is a crucial molecular agonist of the innate immune response and functions to obstruct natural *T*-cell responses and stimulates macrophage activating cytokines, which are essential for mycobacterial clearance (Cooper, 2015). During infection, LM and LAM induce strong pro-inflammatory and anti-inflammatory immune responses, respectively, and the degree of immune activation often correlates to key molecular features of these lipoglycans, including the degree of acylation and length/branching of the mannan backbone (Nigou et al., 2008, Birch et al., 2010). In its tri- and tetra-acylated forms, LM is a potent inducer of pro-inflammatory cytokines, that include TNFα and IL-8 (Vignal et al., 2003, Gilleron et al., 2006). LAM, on the contrary, induces the anti-inflammatory cytokines IL- 10 and TGF-β (Yuan et al., 2019), both of which prime *T*-cells into a Th2 type response, thereby inhibiting phagosome maturation and macrophage activation by suppressing pro-inflammatory cytokines (Domingo-Gonzalez et al., 2016, Correia-Neves et al., 2019). The contrasting activation of the immune response by LM and LAM can be attributed to the presence of a highly branched d-arabinan domain found only in LAM, which is completely absent in LM. In LAM, the d-arabinan polysaccharide masks the pro-inflammatory mannan core that is present and largely identical in both LM and LAM (Nigou et al., 2008). Removal of the d-arabinan domain from LAM by chemical degradation restores the pro-inflammatory nature of LM (Vignal et al., 2003). This effect has been further corroborated in a molecular genetic approach, where a genetic deletion of *MSMEG_2785* (which encodes for arabinofuranosyltransferase C – *aftC*) produces an *M. smegmatis* mutant phenotype, whereby LAM is significantly truncated and possesses pro-inflammatory activity when compared to wild type LAM (Birch et al., 2010). AftC (Rv2673 and Mb2692), is a membrane-bound arabinofuranosyltransferase that is responsible for the 3,5-Ara*f* branching of d-arabinan in both AG and LAM (Birch et al., 2008, Birch et al., 2010, Jankute et al., 2014). Whilst a clean deletion of *aftC* could be achieved in the soil growing saprophytic organism *M. smegmatis* (Birch et al., 2008, Birch et al., 2010)*,* there are no reported studies of *aftC* deletion mutants in the slower growing *M. tuberculosis* or *Mycobacterium bovis* BCG organisms, due to its predicted essentiality (Sassetti et al., 2003).

In this study, we use Clustered Regularly Interspaced Short Palindromic Repeats interference (CRISPRi) to selectively knockdown *aftC* (*Mb2692*) in *M. bovis* BCG and demonstrate that *aftC* depletion causes reduced cell growth, perturbations in cell envelope composition, a truncated LAM and inability to form biofilms. Furthermore*, aftC* depletion results in a *M. bovis* BCG mutant that has reduced infectivity in THP-1 human monocytes, but with significantly increased immunogenicity.

## Materials and methods

### Strains, culture conditions and growth curves

*Escherichia coli* Top10 cells were used in all cloning and vector construction. Cells were grown in Luria- Bertani broth or agar supplemented with appropriate antibiotics at 100 µg/mL kanamycin or 200 µg/mL hygromycin at 37 °C. *M. bovis* BCG was grown in Middlebrook 7H9 medium supplemented with 10 % Middlebrook-Dextrose-Oleic acid-Albumin-Catalase (OADC), 0.05 % Tween 80 and the appropriate antibiotics (50 µg/mL kanamycin or 50 µg/mL hygromycin); or on 7H11 agar supplemented with 10 % OADC and antibiotics at 37 °C.

**Fig. 1:**
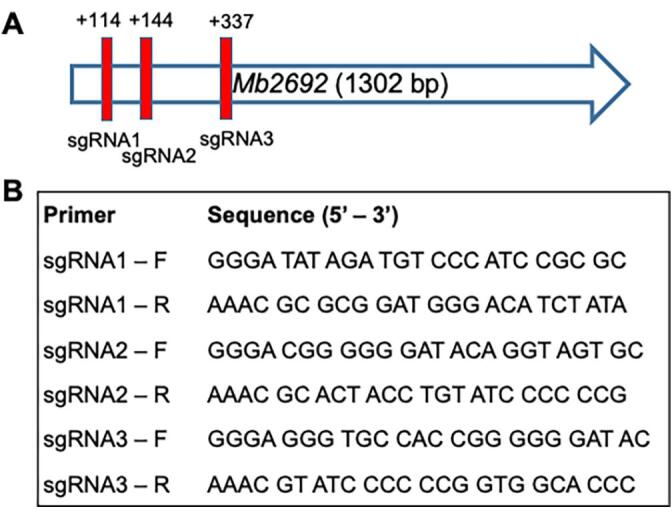
**Generation of *aftC* knockdown strain in *M. bovis* BCG.** (A) Position of sgRNAs used in CRISPRi interference to transcriptionally repress a*ftC* in *M. bovis* BCG. (B) Oligonucleotides used for transcriptional repression of *aftC* in *M. bovis* BCG: Three pairs of oligonucleotides as shown were cloned into pRH2521 under a TetR promoter. Single colonies were grown in the presence of aTC to induce CRISPRi mediated transcriptionally repression of *aftC* in *M. bovis* BCG.

Oligonucleotide primers used for cloning sgRNAs into pRH2521 are listed in Fig. 1. The open reading frames (ORFs) encoding sgRNAs targeting positions +113, +143 and +336 of *M. bovis aftC* (Mb2692) were cloned following standard protocols (Ran et al., 2013). Catalytically inactive or de-active Cas9 (*dCas9*) and the gene-specific single guide RNAs were introduced into *M. bovis* BCG using Tet^R^ regulated plasmids pRH2502 (kanamycin resistance marker and *terRO* controlled *dCas9*) and pRH2521 (hygromycin resistance marker and *tetR* controlled sgRNA – cloned), respectively (Singh et al., 2016). Plasmids were electroporated into electrocompetent *M. bovis* BCG which was prepared by serial washes in 10 % glycerol. Electroporated cells were recovered overnight in 3 mL of 7H9- 10 % OADC- 0.05 % Tween 80 and plated onto 7H11 agar plates supplemented with 10 % OADC, 25 µg/mL kanamycin and 50 µg/mL hygromycin and incubated at 37 °C for 4 weeks.

To monitor the effect of CRISPR interference mediated *aftC* knockdown on growth of *M. bovis* BCG, strains expressing *dCas9* and sgRNA were grown along with a control strain (without the *aft*C targeting sgRNA) in 7H9-OADC-Tween 80-antibiotic medium from a single colony on 7H11-OADC-antibiotic plates at 37 °C. Anhydrotetracycline (aTC) was supplemented in liquid medium at 200 ng/mL every 48 h for continuous induction of sgRNA and *dCas9* expression in CRISPRi experiments. When the optical density (OD) 600 nm of the cultures reached 0.1, the cultures were split into two equal volumes and further incubated with and without aTC supplementation.

### RNA extraction and RT-PCR

*M. bovis* BCG strains were grown in the presence or absence of aTC (BCG-VC [vector control] grown in the presence or absence of aTC was harvested at OD 600 nm of 1.0; BCG-sgRNA3 grown in the absence of aTC and was harvested at OD 600 nm of 0.8; and BCG-sgRNA3 grown in the presence of aTC was harvested at OD 600 nm of 0.3, were centrifuged and resuspended in freshly prepared 5 mg/mL lysozyme solution supplemented with β-mercaptoethanol. Cells were lysed in a bead beater through 2 × 45 *sec* cycles at 6.0 m/*sec*, with an intermediate 5 min cooling step (Fast prep Tissue homogeniser, MP Biomedicals). Cell lysate was supplemented with 3 M sodium acetate (pH 5.2) and subjected to phenol treatment. Samples were thoroughly mixed by inverting the tube and incubated at 65 °C for 5 min. The upper aqueous phase was separated by centrifugation at 17000 × g for 5 min and transferred into a new RNase-free tube and the phenol treatment repeated. The resulting mixture of nucleic acids was cleaned with equal volume of chloroform: isoamyl alcohol (24:1) and transferred into a new tube. RNA was pelleted overnight at −20 °C by the addition of 3 M sodium acetate (pH 5.2) and 100 % ethanol. The RNA was further extracted using 70 % ethanol, air dried, resuspended in DEPC treated RNase free water and quantified using a Nanodrop. Contaminating DNA was removed using TURBO Dnase Enzyme (Thermo Fisher Scientific Inc., Waltham, MA), following the manufacturer’s instructions. The resultant total RNA was used for cDNA synthesis, using the SuperScript™ III First-Strand Synthesis System (Thermo Fisher Scientific Inc., Waltham, MA). Briefly, 2 µg RNA and 2 pmol of gene specific reverse primer of AftC and a housekeeping gene, SigA, 1 µl dNTP mix was made up to 13 µl and heated at 65 °C for 5 min. Further to this mixture, 4 µl of 5X first-strand buffer, 1 µl 0.1 M DTT and 2 µl of Superscript™ III RT were added and incubated at 50 °C for 1 h. The reaction was terminated at the end of 1 h by incubating at 70 °C for 15 min. RNA complementary to the cDNA produced was removed by incubating with RNase A at 37 °C for 20 min. For semi-quantitative estimation of AftC transcripts in the total RNA, PCR was performed using the AftC primer pair (Forward: 5′-GCCGGTCTACCGCGCGGTGCTG −3′; Reverse: 5′-GTGAACACCAGCGTGTTGGTCACGGTC-3′) and standardised against SigA (Forward: 5′-TACGACCAGCACCATCCCGAAAAGGAAG-3′; Reverse: 5′-TCTTCGTCGTCGCCCGAGTCGAGGTC-3′) using the program 98 °C – 3 min; 30 cycles of 98 °C – 10 s, annealing temperature (70 °C for AftC and 69.1 °C for SigA) – 15 s and 72 °C – 30 s; 72 °C – 3 min.

### Microscopy

*M. bovis* BCG strains with induction of *aftC* knock down were grown to an exponential phase. The bacterial pellet was washed thrice with 1X PBS containing 0.05 % Tween 80 and resuspended in 100 µl of PBS with 0.5 % Tween 80. Agarose (1 %) coated microscopy slides were used for immobilisation of bacteria, and the coverslip sealed using clear nail polish. Cell morphology was visualised using a Leica DMRE Widefield Microscope equipped with a 63x/1.25 HC plan Apo objective (oil immersion). Photographs were collected by USB ColourCCD Photometrics MP6 (6 megapixel) camera with a standard RBG filter. Cell length was measured using ImageJ – Fiji software. To enumerate the reduction of cell length, 100 different bacilli from each strain were measured using ImageJ-Fiji software and the results plotted using Graphpad Prism 9.0.1. Student *t*-test was performed between individual groups followed by Mann-Whitney *U* test.

### Biofilm growth conditions, crystal violet and Alcian blue staining

*M. bovis* BCG biofilms were grown as quadruplets in Sauton’s media supplemented with 50 µg/mL kanamycin and 50 µg/mL hygromycin in 12-well plates at 37 °C, 5 % CO_2_ for 5 weeks with 1x PBS along the outer wells to minimise the effect of evaporation. ATC at 200 ng/mL was added carefully every 48 h to maintain CRISPRi induced *aftC* knock down. Once biofilms were matured, photos were captured using a Canon EOS R f/2.8 camera. The biofilms were quantified using a crystal violet assay (Chen et al., 2020). Briefly, biofilms were grown in two biological replicates for each sgRNA3 electroporated *M. bovis* BCG strain. The biofilms were dried, incubated with 0.5 % (w/v) crystal violet at room temperature for 10 min. The cells were washed with water and the stained biofilms air dried. The crystal violet stain from the biofilms was collected in 95 % (v/v) ethanol and quantified at 400 nm using a BMG PHERAstar FS microtitre plate reader. The biofilm polysaccharide content was quantified using an Alcian blue staining assay (Rose et al., 2015). Dried biofilms were stained with 1 % (w/v) Alcian blue 8G in acetic acid pH 2.5 for 20 min at room temperature, washed in water and air dried. The Alcian blue from the biofilms was collected in 33 % (v/v) acetic acid and quantified using a 410 nm in BMG PHERAstar FS microtitre plate reader.

### Extraction of mycobacterial lipoglycans

Planktonic and biofilm cultures were grown and cells collected by centrifugation at 3500 × g for 10 min, washed twice in 1x PBS and transferred into a glass tube. The pellets were resuspended in 50 % ethanol and heated at 90 °C for 3 h. The supernatant containing lipoglycans were collected by centrifugation and transferred into a fresh tube and dried at 50 °C using nitrogen. The ethanol extraction was repeated to ensure the extraction of the residual lipoglycans from the biomass. Proteinase K (2 mg/mL) was prepared in 10 mM Tris (pH 7.5), 20 mM CaCl_2_, and 50 % glycerol was added to the dried extract and incubated at 37 °C for 48 h to ensure complete digestion of proteins in the lipoglycan extract. This was followed by dialysis against endotoxin free water using Pur-a-lyser dialysis kit (MWCO 3.5 kDa) for 48 h and the dialysed material collected into a fresh tube, dried and resuspended in deionised water with 1 % azide to a final concentration of 100 mg/mL.

### Silver staining

LM, LAM and PIMs extracted as above, were separated in a pre-cast Bio-Rad AnyKD gel by SDS-polyacrylamide gel electrophoresis at 200 V, 50 mA for 1 h and stained by a series of solvents: 1) 50 % v/v CH_3_OH, 12 % w/v TCA, 2 % w/v CuCl_2_ (2 hr); 2) 10 % v/v C_2_H_5_OH, 5 % v/v CH_3_COOH (10 min); 3) 0.7 % w/v periodic acid, 40 % v/v C_2_H_5_OH, 5 % v/v CH_3_COOH (10 min); 4) 10 % v/v C_2_H_5_OH (10 min); 5) Deionised water (20 min); 6) 0.1 % w/v AgNO_3_ (10 min); 7) 10 % w/v K_2_CO_3_ (5 min); 8) 2 % w/v K_2_CO_3_ (overnight). The stained gel was visualised using a Bio-Rad imaging system.

### Western blotting

Separated lipoglycans in section 2.5 were transferred onto a nitrocellulose membrane in Tris/Glycine buffer (25 mM Tris, 192 mM Glycine, and 10 % (v/v) methanol) at 25 V, 300 mA for 1.5 h, and blocked for 1 h in 5 % skimmed milk in Tris buffered saline, Tween 20 buffer (20 mM Tris, 150 mM NaCl, and 0.1 % (w/v) Tween 20). The blot was probed using mouse mycobacterial anti-LAM primary antibody, CS-35 (1:1000) overnight at 4 °C, followed by TBST washes and secondary antibody probing using anti-mouse Alexa Fluor™ 633 (Invitrogen) (1:15000) at room temperature for 2 h. Blots were visualised using the LICOR/Odyssey western blot imaging system.

### Lipid extraction and analysis

Post lipoglycan extraction, bacterial pellets were used for the extraction of cell wall associated lipids. Briefly, chloroform/methanol/water (2 mL, 10:10:3, v/v/v) was added to the pellet and heated at 50 °C for 3 h. The supernatant containing lipids was collected by centrifugation at 3000 × g for 10 min and transferred to a new tube. A second chloroform/methanol/water (2 mL, 10:10:3, v/v/v) extraction was repeated to ensure the complete extraction of cell wall associated lipids. The combined lipid extracts were initially washed by mixing for 10 min in 1.75 mL chloroform and 0.75 mL water, where lipids separated into the lower organic layer. The lipids were further washed three times using chloroform/methanol/water (2 mL, 3:47:48, v/v/v) and the washed lipids collected by centrifugation into fresh pre weighed tube and concentrated to 100 mg/mL in chloroform/methanol (2:1, v/v) and used for thin layer chromatography (TLC).

The delipidated cell wall pellet were used for the extraction of cell wall bound lipids, mycolic acids derivatised as methyl esters (MAMEs) or intact bacteria to afford both MAMEs and fatty acid methyl esters (FAMEs). The cell pellet was heated with 2 mL of 5 % tetra-butyl-ammonium hydroxide at 95 °C overnight. This was followed by methylation using 2 mL of water, 4 mL of dichloromethane, and 500 μl of iodomethane for 30 min at room temperature. The cell wall bound MAMEs (and FAMEs) were extracted into a fresh tube and washed three times with water. Lipids were then dried and sonicated in the presence of 4 mL of diethyl ether, and the supernatant containing MAMEs (and FAMEs) transferred to a new tube, dried and redissolved at chloroform: methanol (100 mg, 2:1) and subjected to TLC.

### Infection and cytokine analysis with THP1 cells

THP1 cells were cultured in Gibco RPMI 1640 supplemented media with 10 % heat inactivated fetal bovine serum (HI-FBS) (Sigma-Aldrich) and 1 % Penicillin-Streptomycin (P/S) (Sigma-Aldrich) (growth medium) at 37 °C and 5 % CO_2_. For measuring the infectivity of *M. bovis* BCG *aftC* knock down strains, 0.01 × 10^6^ cells were plated in each well of a 96 well plate and cultured for 72 h at 37 °C and 5 % CO_2_ in RPMI 1640 media supplemented with 20 % HI-FBS, 10 ng/mL PMA and 50 µM β -mercaptoethanol (called Infection medium) for differentiation of monocytes into macrophages. After 72 h, they were infected at a multiplicity of infection (MOI) of 10 with BCG-VC and BCG-sgRNA3 strains grown in the presence or absence of aTC, along with respective controls including wild type *M. bovis* BCG in quintuplets. After infection (4 h), cells were washed 3 times with 1x PBS and incubated with 50 µg/mL gentamycin for 45 min to remove extracellular bacteria within the wells. This was followed by thorough washing using 1x PBS and cell lysis with 1 % (v/v) Triton X-100 in 1x PBS. Cell lysates were diluted and plated onto 7H11 agar plate supplemented with OADC and the respective antibiotics. Plates were incubated for 4 weeks at 37 °C until colonies appeared. For cytokine analysis, 0.1 × 10^6^ THP1 cells were plated in each well of a 48 well plate, differentiated and infected as described above. Post-infection (24 h), supernatants were collected and stored at −80 °C. ELISA was performed according to the manufacturer’ instructions for cytokine analysis.

## Results

### Construction and growth of *M. Bovis* BCG *aftC* knock down strains

The role of non-essential AftC (Ms-AftC) in introducing α(1 → 3) linkages in the arabinan backbone of AG and LAM was demonstrated in *M. smegmatis* (Birch et al., 2008, Birch et al., 2010)*.* AftC is crucial in enhancing arabinan branching and mycolic acid attachment sites (Birch et al., 2008). Here we investigated the effect of transcriptional repression of *aftC* in *M. bovis* BCG (Mb 2692), using CRISPR interference (Choudhary et al., 2015). We cloned three single-guide-RNAs (sgRNAs), sgRNA1, sgRNA2 and sgRNA3, that target three different locations within *aftC* at base-pair (bp) positions of + 113, +143 and + 336, respectively (Fig. 1).

**Fig. 2:**
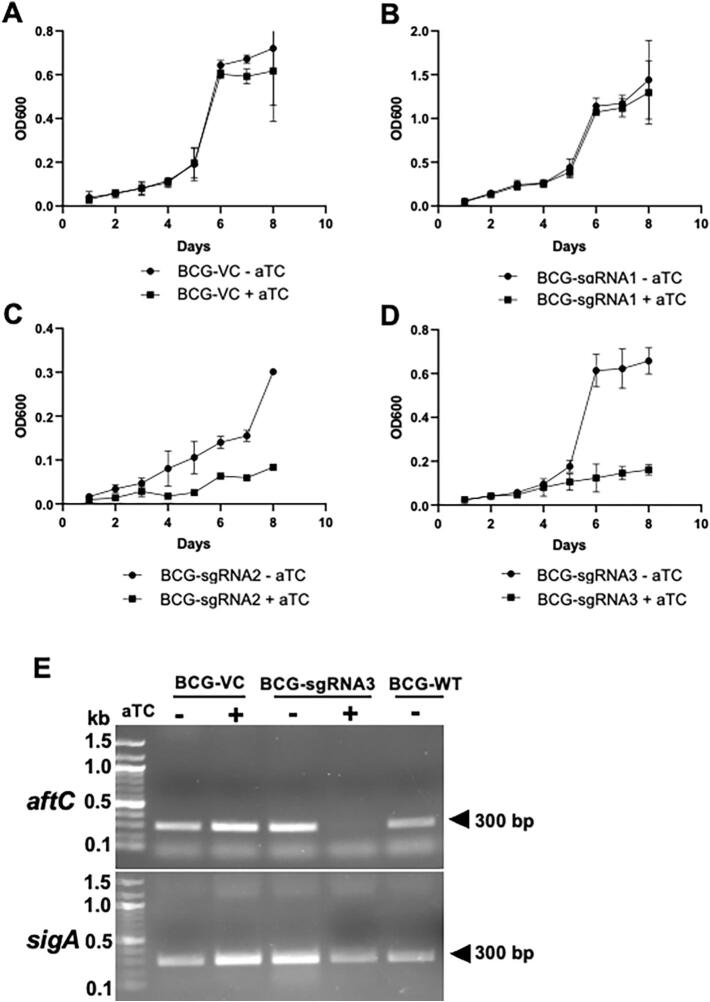
**CRISPRi mediated *aftC* knockdown in *M. bovis* BCG.** Three biological repeats from each strain were grown in the presence or absence of aTC. Absorbance at 600 nm was measured to estimate growth of *M. bovis* BCG when *aftC* knock down is induced by CRISPR interference. (A) BCG-VC which is a control with empty pRH2521 and pRH2502, (B) BCG-sgRNA1, (C) BCG-sgRNA2, and (D) BCG-sg-RNA3 electroporated into *M. bovis* BCG grown both in the presence and absence of aTC. (E) Total RNA extraction, reverse transcription and semi-quantitative PCR using ethidium bromide stained agarose gel electrophoresis were performed on BCG-VC and BCG-sgRNA3 to confirm AftC knock down induced by the addition of aTC in BCG-sgRNA3 when compared to BCG-VC and BCG-WT.

Transformant BCG strains, BCG-VC (empty plasmid transfected *M. bovis* BCG as a control), BCG-sgRNA1, BCG-sgRNA2 and BCG-sgRNA3 were grown as planktonic cultures in liquid medium in the presence and absence of aTC over 8 days to measure their growth kinetics. The optical density (OD), measured at 600 nm revealed normal growth kinetics for BCG-VC in the presence and absence of aTC, confirming the absence of aTC toxicity to mycobacterial cells (Fig. 2A). Similar to BCG-VC, BCG-sgRNA1 was unaffected by aTC induced transcriptional repression of *aftC* (Fig. 2B)*.* In contrast, both BCG-sgRNA2 and BCG-sgRNA3 exhibited a significant growth defect when cultured in the presence of aTC (Fig. 2C, D). This is consistent with the earlier knockout studies in *M. smegmatis* which revealed a growth defect upon *aftC* depletion (Birch et al., 2008). BCG-sgRNA2 has a slower growth rate with an OD 600 reaching 0.3 after 8 days, implying possible leaky expression in the strain affecting its growth in the absence of aTC (Fig. 2C). Due to an inability to attain the required biomass in BCG-sgRNA2 (Fig. 2C) and the absence of a growth defect in BCG-sgRNA1 (Fig. 2B), subsequent analyses were performed with only BCG-sgRNA3, which showed the most significant transcriptional repression of *aftC* in *M. bovis* BCG. Transcriptional repression of *aftC* was confirmed by RNA extraction, reverse transcription and semi-quantitative PCR on BCG-sgRNA3 grown in the presence and absence of aTC and compared with a similarly cultured BCG-VC (Fig. 2E). The house keeping gene *sigA* was used a control to show the significant reduction of *aftC* transcription upon aTC addition in BCG-sgRNA3 (Fig. 2E). The presence of *aftC* transcripts in empty pRH2521 and pRH2502 transformed BCG-VC grown in the presence and absence of aTC confirmed the efficiency of sgRNA3 in transcriptional repression of *aftC*.

**Fig. 3:**
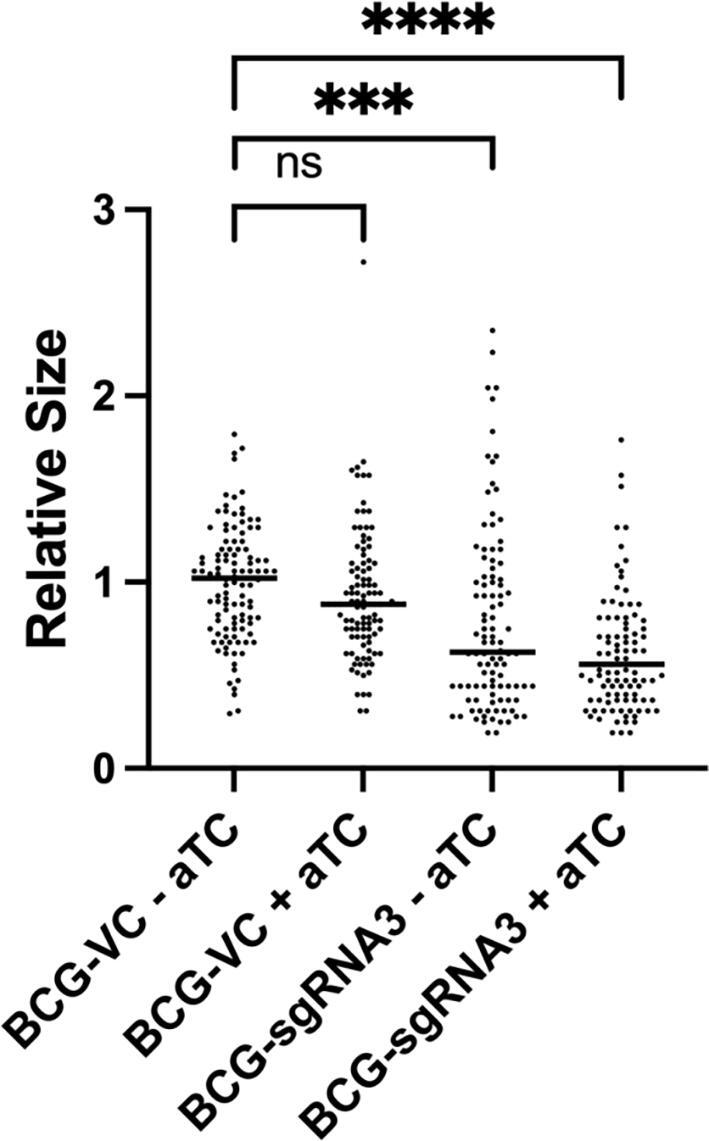
***AftC* repression in *M. bovis* BCG affects cell size.** BCG-VC and BCG-sgRNA3 were grown in the presence or absence of aTC and fixed on 1 % agarose coated microscopy slides and visualised using Leica DMRE Widefield Microscope, cell length measured using ImageJ-Fiji software and plotted using GraphPad Prism 9.0.1 with the BCG-VC -aTC normalised to *M. bovis* BCG WT. Statistical analysis was performed between individual groups using unpaired parametric *t*-test with Welch’s correction (p < 0.05 (*), p < 0.01 (**), p < 0.001 (***), p < 0.0001 (****)). Transcriptional repression of *aftC* has a statistically significant effect on the size of bacilli in *M. bovis* BCG with p < 0.0001.

We investigated the effect of *aftC* knock down on morphology of mycobacterial cells using microscopy. We observed significant reduction in the length of individual bacilli normalised to *M. bovis* BCG WT when comparing BCG-VC to BCG-sgRNA3 (Fig. 3). BCG-VC bacilli appeared to be healthy and of normal length. BCG-sgRNA3 exhibited a heterogeneous population in the absence of aTC with relatively shorter cells, possibly due to a defective cell wall, along with normal sized bacilli. Leaky expression of sgRNA3 and *dCas9* might be responsible for this heterogeneous population. However, BCG-sgRNA3 exhibited a higher number of shorter-sized bacilli, in the presence of aTC. This population had two major types of defective bacilli: a) normal length but a discontinuous cell wall and b) bacilli of significantly smaller cell length. To enumerate this change in cell appearance, the length of 100 different bacilli from each strain were measured (Fig. 3). We observed a statistically significant reduction in cell length of BCG-sgRNA3 grown in the absence of aTC (p < 0.001) as compared to BCG-VC, and this effect was amplified upon addition of aTC (p < 0.0001) (Fig. 3).

### Essentiality of *aftC* in *M. bovis* BCG biofilm formation

**Fig. 4:**
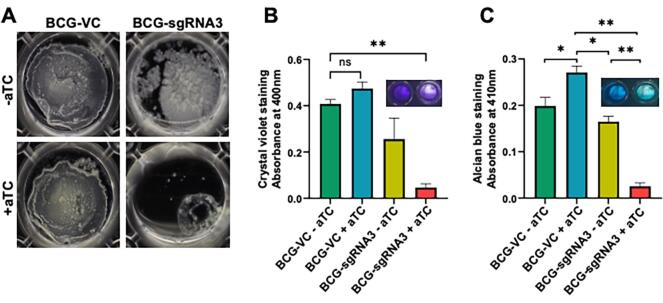
**CRISPRi mediated *aftC* knock down in *M. bovis* BCG affects biofilm formation.** (A) BCG-VC has a stable biofilm formation irrespective of aTC mediated *dCas9* induction. The BCG-sgRNA3 strain of *M. bovis* BCG has a significant reduction in biofilm formation when *aftC* knock down is induced by aTC. (B) Crystal violet assay - biofilm mass in BCG-VC and BCG-sgRNA3 was quantified using a crystal violet assay. (C) Alcian blue staining – acidic polysaccharides in the biofilm matrix were quantified by Alcian blue staining. Statistical analyses were performed between individual groups using unpaired parametric *t*-test with Welch’s correction (p < 0.05 (*), p < 0.01 (**), p < 0.001 (***), p < 0.0001 (****)) in GraphPad Prism 9.0.1 on quadruplet sets of BCG-VC and BCG-sgRNA3 biofilms grown in the absence and presence of aTC. (For interpretation of the references to colour in this figure legend, the reader is referred to the web version of this article.)

We assessed the effect of *aftC* transcriptomic depletion on the ability of *M. bovis* BCG to form biofilms in Sauton’s minimal medium at 37 °C and 5 % CO_2_. While BCG-VC formed mature biofilms in the liquid–air interface within five weeks, BCG-sgRNA3 failed to form mature biofilms (Fig. 4A). Moreover, BCG-VC forms mature biofilm, when grown in the presence of aTC, negating the possibility of aTC induced defective biofilm formation (Fig. 4A). This also highlights that ectopic expression of *dCas9* without sgRNA targeting *aftC,* has no effect on biofilm formation. Additionally, while BCG-sgRNA3 grown in the absence of aTC forms a biofilm, there is a difference in its appearance and texture when compared to biofilms formed by BCG-VC (Fig. 4A). BCG-sgRNA3 lacks the organised ridges and troughs characteristic of mycobacterial biofilms and instead forms a cluster of multiple microcolonies aggregated together (Fig. 4A). The reduction in biofilm formation seen in BCG-sgRNA3 grown in the absence of aTC can be attributed to the leaky expression of sgRNA3 and *dCas9* with defective bacilli, as confirmed by microscopy (Fig. 3). However, defects in BCG-sgRNA3 biofilm formation were amplified when grown in the presence of aTC inducing CRISPR interference mediated transcriptional repression of *aftC*. Biofilms did not form upon *aftC* repression (Fig. 4A, B). When compared to BCG-VC grown in the presence of aTC there is a 2-fold reduction in biofilm formation of BCG-sgRNA3 grown in the absence of aTC and a 10-fold reduction in biofilm biomass in BCG-sgRNA3 grown in the presence of aTC (Fig. 4B). This confirms that *M. bovis* BCG *aftC* plays a significant role in biofilm formation.

**Fig. 5:**
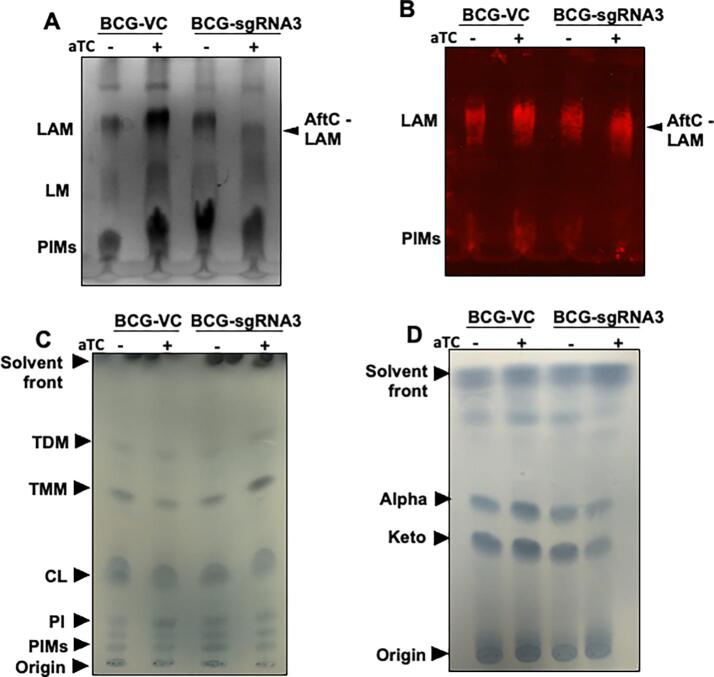
**Cell wall lipoglycan and lipid analysis of CRISPRi mediated *aftC* knock down *M. bovis* BCG biofilms.** (A-B) Extracted cell wall lipoglycans from biofilm cultures were separated on SDS-PAGE gels and analysed by silver staining (A) and Western blot (B) using a CS-35 antibody. (C) Cell wall associated lipids were extracted and analysed by 1-D TLC and stained using molybdophosphoric acid (MPA) to visualise lipids. (D) MAMEs were extracted and analysed by TLC and stained using MPA.

As *aftC* is involved in the assembly of mycobacterial d-arabinan of both AG and LAM, polysaccharides were quantified within *M. bovis* BCG biofilms, the extracellular matrix, and those attached to the floating cell surfaces within the biofilm, using Alcian Blue. Dye retained by the samples, measured by absorbance at 410 nm is an index of the abundance of polysaccharides in the biofilm matrix (Fig. 4C). Alcian blue staining between BCG- VC and BCG-sgRNA3 were comparable, when both were grown in the absence of aTC. However, a 1.5-fold higher stain was retained in BCG-VC samples, when grown in the presence of aTC, compared to the same strain grown in the absence of aTC (Fig. 4C). In BCG-VC, which lacks sgRNA targeting *aftC*, excess aTC accumulated in sub-lethal levels might have caused this increase in polysaccharide levels within its biofilm matrix. However, the minimum inhibitory concentration (MIC) of aTC in *M. smegmatis* planktonic cultures is around 1 µg/mL (Ehrt et al., 2005) and in this study, we use aTC at a concentration that is 5-fold below its MIC in *M. bovis* BCG biofilms. Therefore, there must be no possible aTC induced toxicity contributing to this effect. Furthermore, similar aTC induced toxicity was not seen in planktonic cultures (Fig. 2, Fig. 3) and no corroborating evidence was found in the biochemical characterisation of the biofilm matrix itself (Fig. 5). BCG-sgRNA3 biofilms grown in the presence of aTC showed a 7-fold reduction in acidic polysaccharide levels as compared to the same strain grown in the absence of aTC (Fig. 4C). Although, there has been a slight increase in overall polysaccharide level in BCG-VC biofilms, when grown in the presence of aTC, this effect is not observed in BCG-sgRNA3. Alcian blue was retained 10-fold lower in BCG-sgRNA3 biofilms grown in the presence of aTC as compared to BCG-VC biofilms grown in the presence of aTC (Fig. 4C). This data confirms the role of *aftC* in the generation and assembly of mycobacterial cell envelope polysaccharides within the biofilm matrix of *M. bovis* BCG.

### Characterisation of lipoglycans extracted from an *aftC* knock down *M. bovis* BCG strain

**Fig. 6:**
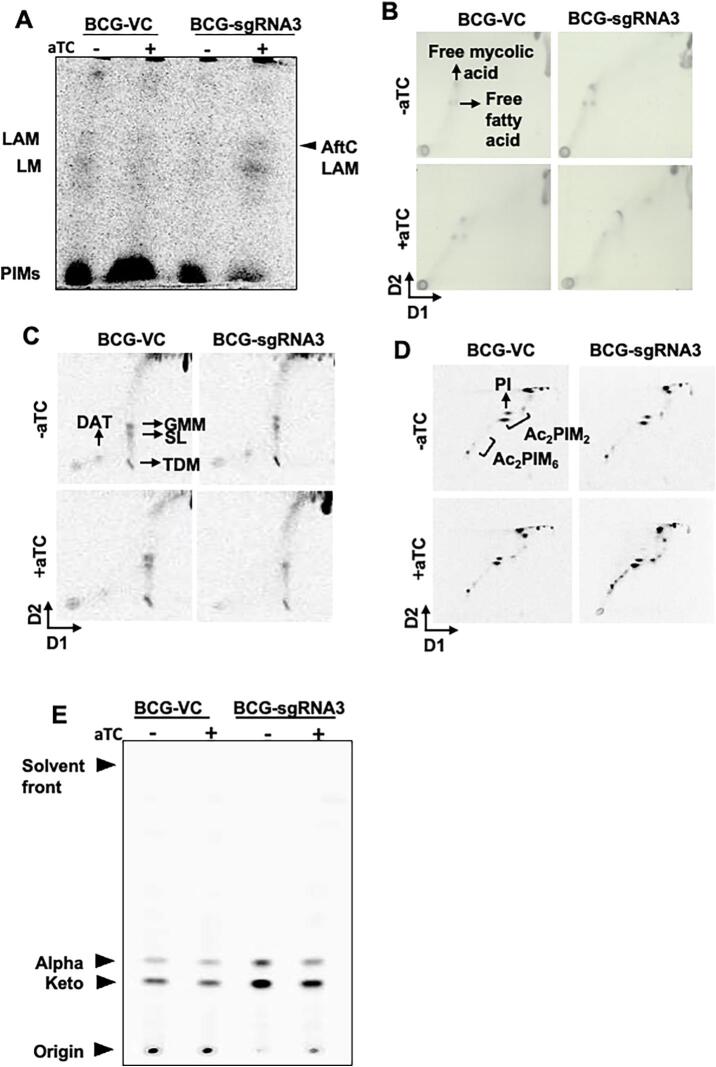
**Cell wall lipoglycan and lipid analysis of CRISPRi mediated *aftC* knock down in *M. bovis* BCG planktonic cultures.** (A) ^14^C lipoglycans were separated by SDS-PAGE gel and visualised using autoradiography. (B, C, D and E) Extracted lipids and MAMES were separated using TLC and visualised using either MPA staining or a phosphor screen autoradiography.

Lipoglycan composition within biofilm cultures of *aftC* repressed *M. bovis* BCG were analysed using silver staining (Fig. 5A) and Western blot analysis using the CS-35 antibody which specifically binds to the terminal d-arabinan motifs of LAM (Fig. 5B). While aTC had no effect on the lipoglycan composition of BCG-VC, BCG-sgRNA3 produced an intermediary sized LAM when grown in the presence of aTC (labelled as ‘AftC-LAM’) (Fig. 5 A and B). Western blot analysis using CS-35 confirmed this reduction in size of AftC-LAM in BCG-sgRNA3 (Fig. 5B). The planktonic cell wall composition of *aftC* repressed *M. bovis* BCG was further analysed by ^14^C radiolabeling and autoradiography. Similar to the lipoglycan analysis, BCG-sgRNA3 exhibited an intermediary sized LAM upon aTC induced repression of *aftC,* while BCG-VC exhibited no change in its lipoglycan profile upon aTC addition (Fig. 6A). This is consistent with *aftC* knockout studies in *M. smegmatis*, where similar intermediary sized LAM was observed upon *aftC* depletion that was restored upon AftC complementation (Birch et al., 2008, Birch et al., 2010). Densitometry revealed no difference in the amount of LAM produced upon *aftC* repression. LAM produced by BCG-VC was in comparable in abundance to AftC-LAM.

### Cell envelope lipid profile of *aftC* knock down *M. bovis* BCG

To verify the effect of *aftC* repression in *M. bovis* BCG on cell envelope lipid composition, cell wall associated lipids were extracted from both biofilm cultures (Fig. 5C) and ^14^C radiolabeled planktonic cultures (Fig. 6B, C, D) and analysed by TLC. Other than the accumulation of TMM in *aftC* transcriptionally repressed BCG-sgRNA3 (Fig. 5C), no significant changes in cell wall associated lipids were observed. Similarly, cell wall bound MAMEs extracted from delipdated biofilm cultures (Fig. 5D) and ^14^C radiolabeled planktonic cultures (Fig. 6E) were analysed by TLC. A reduction of MAMEs was observed (Fig. 5D and Fig. 5E) in BCG-sgRNA3 strains in the presence of aTC. Earlier *aftC* knockout studies in *M. smegmatis* revealed reduction in AG esterified mycolic acids and increase in TDM production upon *aftC* depletion (Birch et al., 2008). Our studies show a similar change in AG esterified mycolic acids and cell wall associated lipids (TMM) upon *aftC* repression in BCG- sgRNA3 in its planktonic and biofilm cultures.

### Infectivity of *aftC* knock down *M. bovis* BCG strains

**Fig. 7:**
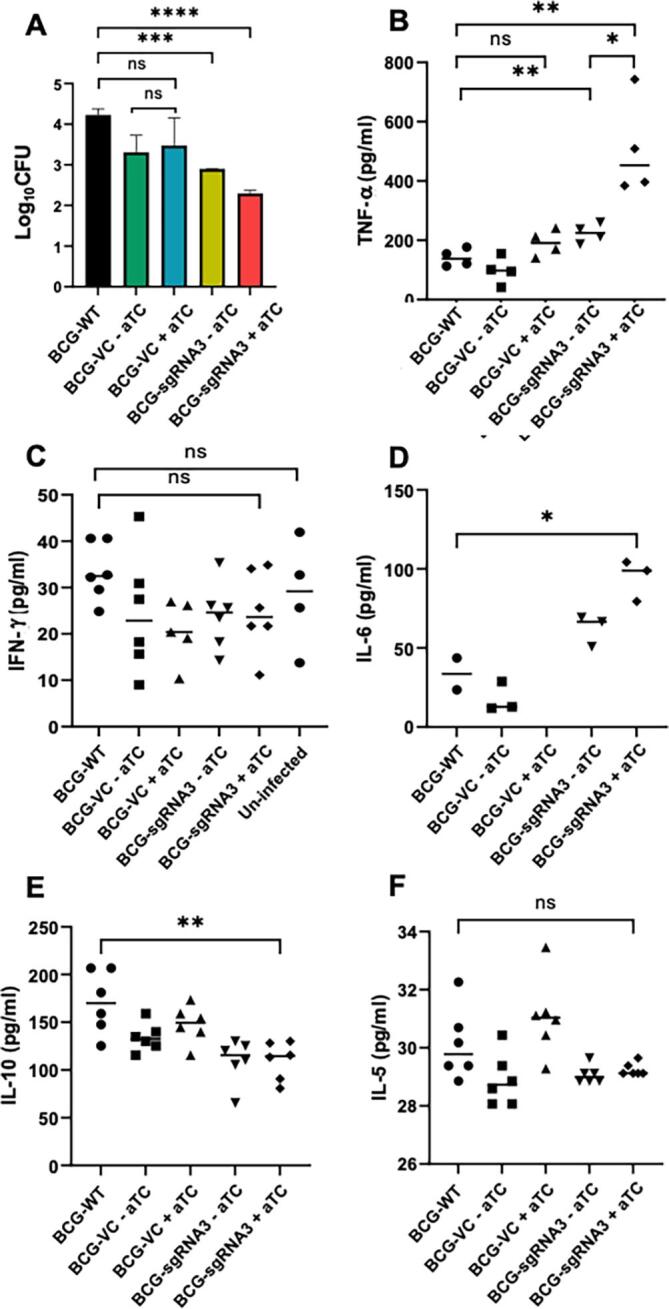
***aftC* repressed *M. bovis* BCG has reduced infectivity and increased immunogenicity in THP1 cells.** (A) THP1s were infected with BCG-VC and BCG-sgRNA3 and cell lysates diluted and plated onto 7H11 agar plates with appropriate supplements. (B) *aftC* repressed BCG-sgRNA3 produced a 3-fold higher level of TNF- α in comparison to BCG-WT. (C) IFN-γ levels were similar in both *aftC* repressed BCG- sgRNA3 and BCG-WT. (D) IL-6 levels were measured and there is statistically significant 2-fold increase in IL-6 level produced in *aftC* repressed BCG-sgRNA3. (E) IL-10 was measured and was reduced 2-fold in *aftC* repressed BCG-sgRNA3 when compared to BCG- WT. (F) IL-5 levels were comparable in *aftC* repressed BCG-sgRNA3 and BCG-WT and there is no statistically significant difference. Statistical analysis was performed between individual groups in GraphPad Prism 9.0.1 using unpaired parametric *t*-test with Welch’s correction (p < 0.05 (*), p < 0.01 (**), p < 0.001 (***), p < 0.0001 (****)).

We next examined the effect of *aftC* repression on the pathogenesis of *M. bovis* BCG in a THP1 cell line. Infection studies were performed to evaluate macrophage uptake and intracellular survival of BCG-sgRNA3 and BCG-VC strains grown in the presence and absence of aTC. THP1s were infected at multiplicity of infection (MOI) of 10 with BCG-sgRNA3 and BCG- VC grown in the presence and absence of aTC. Intracellular bacterial survival was measured 4 h post infection by plating serial dilutions of cell lysates and enumerating CFUs 4 weeks post incubation at 37 °C. Consistent with previous studies (Fukuda et al., 2013) BCG-sgRNA3 grown in the presence of aTC is more sensitive to macrophage killing. BCG-VC grown in the presence or absence of aTC is comparable to wild type *M. bovis* BCG in intracellular survival within macrophages (Fig. 7A). Similar to previous results (Fig. 3, Fig. 4), BCG-sgRNA3 grown in the absence of aTC showed reduced intracellular survival in THP1s (Fig. 7A). This effect is amplified in *aftC* repressed BCG-sgRNA3 grown in the presence of aTC (Fig. 7A). This data confirms the role of LAM in macrophage uptake and intracellular survival of *M. bovis* BCG in THP1s which is hindered in *aftC* repressed *M. bovis* BCG.

### Immunogenicity of *aftC* knock down *M. bovis* BCG strains

LAM in contrast to LM is a potent anti-inflammatory lipoglycan, owing to the presence of bulky large arabinan domain masking the “bioactive” mannan core (Vignal et al., 2003, Quesniaux et al., 2004, Doz et al., 2007, Nigou et al., 2008). In *aftC* repressed *M. bovis* BCG which contains an unbranched truncated ‘AftC-LAM’ this “bioactive” mannan core is exposed. As a result of this, we have seen an increase in pro-inflammatory cytokine production and a decrease in anti-inflammatory cytokine production from THP1s infected with *aftC* repressed BCG-sgRNA3 when compared to BCG-WT (Fig. 7B-F). TNF-α is a major pro-inflammatory cytokine produced by macrophages during mycobacterial infection (Flynn and Chan, 2001, Wajant et al., 2003, Domingo-Gonzalez et al., 2016). We observe a significant 3-fold upregulation of TNF- α production from THP1 cells infected with *aftC* repressed BCG-sgRNA3 in the presence of aTC as compared to BCG-WT.

In contrast to TNF- α which is primarily produced by macrophages, IFN-γis initially produced by *T*-cells which then affect the migratory and functional capacity of phagocytes including macrophages, NK-cells and dendritic cells (Cooper, 2009, Greenlund et al., 1994, Kovarik et al., 1998). These phagocytes once stimulated, produce IFN-γ themselves to maintain a continual expression of this crucial pro-inflammatory cytokine (Schroder et al., 2004, Reed et al., 2008). Since there is no prior stimulation, THP1s used in the assay failed to produce statistically significant levels of IFN-γ upon infection with BCG-WT or *aftC* repressed BCG-sgRNA3 (Fig. 7C). IL-6 is another inflammatory cytokine produced in response to TNF- α (Shalaby et al., 1989) and is crucial in mycobacterial clearance as mice deficient of IL-6 had increased susceptibility to mycobacterial infection (Appelberg et al., 1994, Ladel et al., 1997). *AftC* repressed BCG-sgRNA3 produced a statistically significant 3-fold increased production of IL- 6 in comparison to BCG-WT (Fig. 7D). IL-12, IL-12p70, CD-40, IL-1b, IL-17, IL-17E were tested for their cytokine levels and no significant difference was observed between *aftC* repressed BCG- sgRNA3 and BCG-WT. IL-10 is a crucial regulatory cytokine produced by all immune cells to limit harmful excessive inflammatory immune response (Vieira et al., 1991, Redford et al., 2011). In the context of *M. tuberculosis*, IL-10 production correlates with increased bacterial burden and severe TB (Eum et al., 2008, Kumar et al., 2015, Tebruegge et al., 2015). In macrophages, IL-10 can block phagosome maturation and inhibit IFN-γ mediated macrophage activation thereby creating a niche for mycobacterial replication (Oswald et al., 1992, O'Leary et al., 2011). THP1s when infected with *aftC* repressed BCG-sgRNA3, have shown 1.5-fold reduction in IL-10 production in comparison with BCG-WT thereby promoting enhanced mycobacterial clearance of the cell wall-defective strain as compared to the wild-type strain (Fig. 7E). IL-4, IL-13 and IL-5 (Fig. 7F) are other regulatory cytokines having similar regulatory function to suppress the production of pro-inflammatory cytokines to enable tissue repair and maintain homeostasis (Mosmann et al., 1986, Appelberg et al., 1992, Powrie et al., 1993). Although there is no statistically significant downregulation of these regulatory cytokines in THP1s when infected with *aftC* repressed BCG-sgRNA3 or BCG-WT, we cannot ignore the role of other innate immune cells in host-mycobacterial interactions. Therefore, further *in vivo* infection assays using mice models can shed further light on the immune stimulatory properties of *aftC* repressed BCG- sgRNA3.

Overall the data confirms the enhanced immunogenicity of *aftC* repressed *M. bovis* BCG owing to the shorter, unbranched ‘AftC- LAM’ with the exposed “bioactive” mannan core within its cell envelope. We have observed an increased pro-inflammatory cytokine production including TNF-α and IL-6 and reduced anti-inflammatory IL-10 production in *aftC* repressed *M. bovis* BCG in comparison to BCG-WT. This is consistent with the earlier studies using purified lipoglycans extracted from an *aftC M. smegmatis* knockout which exhibited an increased pro-inflammatory activity (Birch et al., 2010).

## Discussion

Traditional gene depletion studies targeting essential genes, involves the generation of a merodiploid strain, with regulated expression of targeted gene by an inducible promoter in a strain in which the native expression has been inhibited (Birch et al., 2008). However, in slow growing bacteria such as *M. bovis* BCG and *M. tuberculosis*, with a generation time of 20 h, this method becomes lengthy and time consuming. A recently developed novel transcriptional repression system, involving CRISPRi presents an alternative approach to gene repression with numerous advantages over the conventional gene deletion methods (Chhotaray et al., 2018, Choudhary et al., 2015, Choudhary et al., 2016, Qi et al., 2013, Rock et al., 2017). In this approach, a 20 base pair short guide RNA (sgRNA) complementary to the gene of interest, recruits a catalytically inactive Cas9 (*dCas9*) protein to the target gene and induces transcriptional repression *via* steric inhibition of either transcription initiation or elongation, resulting in the lower levels of RNA of the gene of interest (Bikard et al., 2013, Qi et al., 2013). One major advantage of this approach is the ability to control the level of transcriptional repression, since the expression of both sgRNA and dCas9 can be controlled by the inducible Tet repressor (Tet^R^) regulated promoter thereby achieving a range of inhibition of expression of the target gene (Singh et al., 2016). A recent report demonstrated CRISPRi in *M. tuberculosis* with sustained knockdown of several essential gene with less off target effects in *M. tuberculosis* (Singh et al., 2016). CRISPRi is also multiplexed which is particularly important in slow growing mycobacteria, such as *M. bovis* and *M. tuberculosis,* thus reducing time consuming serial genetic manipulations in methods such as specialised transduction (Rock et al., 2017). Considering these advantages, we generated a *M. bovis* BCG cell wall mutant strain by CRISPRi-mediated *aftC* transcriptional repression. In this study, we also describe an optimised system for CRISPRi mediated gene silencing in biofilm cultures of mycobacteria. We further characterised the phenotypic and biochemical consequences of *aftC* repression in planktonic and biofilm cultures of *M. bovis* BCG. Further, we demonstrated the reduced infectivity and increased immuno-stimulatory properties of *aftC* repressed *M. bovis* BCG in THP1 cells.

Earlier AftC deletion studies in *M. smegmatis* and *C. glutamicum* revealed its biosynthetic role as an α (1 → 3) branching arabinofuranosyltransferase of d-arabinan within the mycobacterial cell envelope (Birch et al., 2008). *AftC* is a crucial priming enzyme responsible for the addition of the first 3,5- arabinofuranosyl branching residue within both arabinogalactan (Birch et al., 2008) and LAM (Birch et al., 2010), which are then extended by other arabinofuranosyltransferases to form long arabinan chains (Jankute et al., 2015). The non-reducing terminal of the arabinan unit serves as an attachment site for mycolic acids (McNeil et al., 1991) and the *aftC* deletion strain with unbranched arabinan units within arabinogalactan and LAM contained fewer cell wall bound mycolic acids (Birch et al., 2008). These changes in the cell envelope composition seen upon *aftC* deletion significantly affected acid-fastness, viability (Birch et al., 2008), antibiotic sensitivity (rifampicin, chloramphenicol, and ethambutol) and cytokine stimulatory profile of *aftC* depleted *M. smegmatis* (Birch et al., 2010).

Here, transcriptional repression of *aftC* in *M. bovis* BCG resulted in pleiotropic variations of cell phenotype. *AftC* depletion significantly lowered planktonic growth rate (Fig. 2) and biofilm formation (Fig. 4) and a smaller cell size (Fig. 3). Although reduction in biofilm formation (Fig. 4) was independent of cell lysis (Fig. 2), altered biofilm morphology was potentially mediated by the reduction in extracellular acidic polysaccharides, including LAM (Fig. 4). Moreover, consistent with earlier studies in *M. smegmatis* (Birch et al., 2010), *aftC* repression resulted in generation of a truncated intermediary sized LAM (termed ‘AftC-LAM’) in both planktonic and biofilm cultures of *M. bovis* BCG (Fig. 5A-B, 6A).

Infection studies in THP1 cells using *aftC* repressed *M. bovis* BCG revealed reduced bacterial uptake and intracellular survival of mutant strains as compared to wild type *M. bovis* BCG (Fig. 7A). This is a direct consequence of ‘AftC-LAM’ present within the cell envelope of *aftC* repressed *M. bovis* BCG, as no other significant alterations to the cell envelope composition could be observed (Fig. 5, Fig. 6). C-type lectins are essential pattern recognition receptors expressed on the surface of macrophages, dendritic cells and neutrophils which recognise LAM through their carbohydrate recognition domains (Drickamer and Taylor, 2015, Schnaar, 2015). LAM is a crucial immunomodulator and an important pathogen associated molecular pattern imperative for the initial interaction between mycobacteria and innate immune cells. Reduced bacterial uptake seen in *aftC* repressed *M. bovis* BCG is contributed by the defective, short AftC-LAM (Fig. 6A). As it has reduced arabinan branching and mycolic acid attachment sites, AftC-LAM therefore has fewer mannose capping motifs (Birch et al., 2008) which are also crucial for immunomodulation. Mannose caps on LAM are essential for endocytic pathways within innate immune cells, especially for mannose receptor and DC-SIGN which bind to mannose caps on LAM resulting in efficient internalisation of mycobacteria (Maeda et al., 2003, Kang et al., 2005). *AftC* repressed *M. bovis* BCG displaying ‘AftC-LAM’ and fewer mannose caps therefore displayed reduced bacterial uptake and intracellular survival in THP1s.

Cytokine stimulatory activity of *aftC* repressed *M. bovis* BCG is consistent with the previous studies in *M. smegmatis* (Birch et al., 2010). ‘AftC-LAM’ in *aftC* repressed *M. bovis* BCG has an exposed ‘bioactive’ mannan core that was otherwise masked by the bulky arabinan domain as in wild type LAM (Vignal et al., 2003, Quesniaux et al., 2004, Doz et al., 2007, Nigou et al., 2008). As a result of this, we observe an increased pro-inflammatory cytokine production, such as TNF- α and IL-6 and reduced anti-inflammatory IL-10 production in THP1s infected with *aftC* repressed *M. bovis* BCG in comparison to BCG-WT (Fig. 7B-F). This is consistent with earlier studies in *M. smegmatis* where purified LAM extracted from *aftC* knockout *M. smegmatis* showed increased production of pro-inflammatory cytokines TNF-α and IL-8 in THP1s as compared to *M. smegmatis* wild type LAM (Birch et al., 2010). Other crucial cytokines including IFN-γ, IL-1b, IL-12, IL-4 and IL-17 were measured (Fig. 7). However, considering our infection assay was designed to identify cytokines produced by THP1s independent of other cells of innate immunity, an incomplete complete picture of cell mediated immunity induced by *aftC* repressed *M. bovis* BCG persists. This is particularly important because IFN-γ is initially produced by *T*-cells, leading to activation of macrophages and other phagocytes (Greenlund et al., 1994, Kovarik et al., 1998) which can then produce IFN-γ and other crucial cytokines themselves to enhance bacterial clearance (Schroder et al., 2004, Reed et al., 2008). Since there was no prior stimulation of THP1s used in the assay, we were unable to see the effect of *aftC* repressed BCG-sgRNA3 on other crucial cytokines (Fig. 7). Therefore, further *in vivo* experiments are essential to establish the immune stimulatory properties of this immunogenic *aftC* repressed BCG-sgRNA3 strain.

In this study, we have established that *aftC* repressed *M. bovis* BCG results in the generation of short, truncated LAM within its cell envelope in both planktonic and biofilm cultures. Moreover, infection and cytokine studies in THP1 cells revealed reduced virulence and enhanced immunogenic properties of *aftC* repressed *M. bovis* BCG. Additionally, we confirmed CRISPRi to be a powerful technology for mycobacterial gene manipulation enabling facile manipulation of essential genes.

## CRediT authorship contribution statement

**Bala T.S.A. Madduri:** Conceptualization, Writing – original draft, Writing – review & editing, Visualization. **Lauren Allen:** . **Stephen C. Taylor:** Writing – original draft. **Gurdyal S. Besra:** Conceptualization, Writing – original draft, Writing – review & editing. **Luke J. Alderwick:** Conceptualization, Writing – original draft, Writing – review & editing, Visualization.

## Declaration of Competing Interest

The authors declare the following financial interests/personal relationships which may be considered as potential competing interests: Gurdyal Singh Besra reports financial support was provided by Medical Research Council. Bala T. S. A. Madduri reports financial support was provided by Darwin Trust of Edinburgh. Gurdyal Singh Besra reports a relationship with Microbiology Society that includes: travel reimbursement.

## References

[b0005] Alderwick L.J., Harrison J., Lloyd G.S., Birch H.L. (2015). The Mycobacterial Cell Wall-Peptidoglycan and Arabinogalactan. Cold Spring Harb. Perspect. Med..

[b0010] Appelberg R., Orme I.M., Pinto de Sousa M.I., Silva M.T. (1992). In vitro effects of interleukin-4 on interferon-gamma-induced macrophage activation. Immunology.

[b0015] Appelberg R., Castro A.G., Pedrosa J., Minóprio P. (1994). Role of interleukin-6 in the induction of protective T cells during mycobacterial infections in mice. Immunology.

[b0020] Bikard D., Jiang W., Samai P., Hochschild A., Zhang F., Marraffini L.A. (2013). Programmable repression and activation of bacterial gene expression using an engineered CRISPR-Cas system. Nucleic Acids Res..

[b0025] Birch H.L., Alderwick L.J., Bhatt A., Rittmann D., Krumbach K., Singh A., Bai Y., Lowary T.L., Eggeling L., Besra G.S. (2008). Biosynthesis of mycobacterial arabinogalactan: identification of a novel alpha (1–>3) arabinofuranosyltransferase. Mol. Microbiol..

[b0030] Birch H.L., Alderwick L.J., Appelmelk B.J., Maaskant J., Bhatt A., Singh A., Nigou J., Eggeling L., Geurtsen J., Besra G.S. (2010). A truncated lipoglycan from mycobacteria with altered immunological properties. PNAS.

[b0035] Chen S., Teng T., Wen S., Zhang T., Huang H. (2020). The aceE involves in mycolic acid synthesis and biofilm formation in Mycobacterium smegmatis. BMC Microbiol..

[b0040] Chhotaray C., Tan Y., Mugweru J., Islam M.M., Adnan Hameed H.M., Wang S., Lu Z., Wang C., Li X., Tan S., Liu J., Zhang T. (2018). Advances in the development of molecular genetic tools for Mycobacterium tuberculosis. J. Genet Genom..

[b0045] Choudhary C., Lunge A., Agarwal N. (2016). Strategies of genome editing in mycobacteria: Achievements and challenges. Tuberculosis (Edinb)..

[b0050] Choudhary E., Thakur P., Pareek M., Agarwal N. (2015). Gene silencing by CRISPR interference in mycobacteria. Nat. Commun..

[b0055] Cooper A.M. (2009). Cell-mediated immune responses in tuberculosis. Annu. Rev. Immunol..

[b0060] Correia-Neves M., Sundling C., Cooper A., Källenius G. (2019). Lipoarabinomannan in Active and Passive Protection against Tuberculosis. Front. Immunol..

[b0065] Domingo-Gonzalez R., Prince O., Cooper A., Khader S.A. (2016). Cytokines and Chemokines in Mycobacterium tuberculosis Infection. Microbiology. Spectrum.

[b0070] Doz E., Rose S., Nigou J., Gilleron M., Puzo G., Erard F., Ryffel B., Quesniaux V.F. (2007). Acylation determines the toll-like receptor (TLR)-dependent positive versus TLR2-, mannose receptor-, and SIGNR1-independent negative regulation of pro-inflammatory cytokines by mycobacterial lipomannan. J. Biol. Chem..

[b0075] Drickamer K., Taylor M.E. (2015). Recent insights into structures and functions of C- type lectins in the immune system. Curr. Opin. Struct. Biol..

[b0080] Ehrt S., Guo X.V., Hickey C.M., Ryou M., Monteleone M., Riley L.W., Schnappinger D. (2005). Controlling gene expression in mycobacteria with anhydrotetracycline and Tet repressor. Nucleic Acids Res..

[b0085] Eum S.Y., Jeon B.Y., Min J.H., Kim S.C., Cho S., Park S.K., Cho S.N. (2008). Tumor necrosis factor-alpha and interleukin-10 in whole blood is associated with disease progression in pulmonary multidrug-resistant tuberculosis patients. Respiration.

[b0090] Flynn J.L., Chan J. (2001). Immunology of tuberculosis. Annu. Rev. Immunol..

[b0095] Fukuda T., Matsumura T., Ato M., Hamasaki M., Nishiuchi Y., Murakami Y., Maeda Y., Yoshimori T., Matsumoto S., Kobayashi K., Kinoshita T., Morita Y.S. (2013). Critical roles for lipomannan and lipoarabinomannan in cell wall integrity of mycobacteria and pathogenesis of tuberculosis. mBio.

[b0100] Gilleron M., Nigou J., Nicolle D., Quesniaux V., Puzo G. (2006). The acylation state of mycobacterial lipomannans modulates innate immunity response through toll-like receptor 2. Chem Biol..

[b0105] Greenlund A.C., Farrar M.A., Viviano B.L., Schreiber R.D. (1994). Ligand-induced IFN gamma receptor tyrosine phosphorylation couples the receptor to its signal transduction system (p91). EMBO J..

[b0110] Jankute M., Cox J.A., Harrison J., Besra G.S. (2015). Assembly of the Mycobacterial Cell Wall. Ann. Rev. Microbiol..

[b0115] Jankute M., Alderwick L.J., Noack S., Veerapen N., Nigou J., Besra G.S. (2017). Disruption of Mycobacterial AftB Results in Complete Loss of Terminal β(1 → 2) Arabinofuranose Residues of Lipoarabinomannan. ACS Chem. Biol..

[b0120] Jankute, M., Grover, S., Birch, H.L., Besra, G.S., 2014. Genetics of Mycobacterial Arabinogalactan and Lipoarabinomannan Assembly. Microbiology spectrum, 2(4), MGM2–2013. https://doi.org/10.1128/microbiolspec.MGM2-0013-2013. 10.1128/microbiolspec.MGM2-0013-2013 26104198

[b0125] Kang P.B., Azad A.K., Torrelles J.B., Kaufman T.M., Beharka A., Tibesar E., DesJardin L.E., Schlesinger L.S. (2005). The human macrophage mannose receptor directs Mycobacterium tuberculosis lipoarabinomannan-mediated phagosome biogenesis. J. Exp. Med..

[b0130] Kovarik P., Stoiber D., Novy M., Decker T. (1998). Stat1 combines signals derived from IFN-gamma and LPS receptors during macrophage activation. EMBO J..

[b0135] Kumar N.P., Moideen K., Banurekha V.V., Nair D., Sridhar R., Nutman T.B., Babu S. (2015). IL-27 and TGFβ mediated expansion of Th1 and adaptive regulatory T cells expressing IL-10 correlates with bacterial burden and disease severity in pulmonary tuberculosis. Immun. Inflamm Dis..

[b0140] Ladel C.H., Blum C., Dreher A., Reifenberg K., Kopf M., Kaufmann S.H. (1997). Lethal tuberculosis in interleukin-6-deficient mutant mice. Infect. Immun..

[b0145] Maeda N., Nigou J., Herrmann J.L., Jackson M., Amara A., Lagrange P.H., Puzo G., Gicquel B., Neyrolles O. (2003). The cell surface receptor DC-SIGN discriminates between Mycobacterium species through selective recognition of the mannose caps on lipoarabinomannan. J. Biol. Chem..

[b0150] McNeil M., Daffe M., Brennan P.J. (1991). Location of the mycolyl ester substituents in the cell walls of mycobacteria. J. Biol. Chem..

[b0155] Mosmann T.R., Cherwinski H., Bond M.W., Giedlin M.A., Coffman R.L. (1986). Two types of murine helper T cell clone. I. Definition according to profiles of lymphokine activities and secreted proteins. J. Immunol..

[b0165] Nigou J., Vasselon T., Ray A., Constant P., Gilleron M., Besra G.S., Sutcliffe I., Tiraby G., Puzo G. (2008). Mannan chain length controls lipoglycans signaling via and binding to TLR2. J. Immunol..

[b0170] Ojha A.K., Baughn A.D., Sambandan D., Hsu T., Trivelli X., Guerardel Y., Alahari A., Kremer L., Jacobs W.R., Hatfull G.F. (2008). Growth of Mycobacterium tuberculosis biofilms containing free mycolic acids and harbouring drug-tolerant bacteria. Mol. Microbiol..

[b0175] O'Leary S., O'Sullivan M.P., Keane J. (2011). IL-10 blocks phagosome maturation in mycobacterium tuberculosis-infected human macrophages. Am. J. Respir. Cell Mol. Biol..

[b0180] Oswald I.P., Wynn T.A., Sher A., James S.L. (1992). Interleukin 10 inhibits macrophage microbicidal activity by blocking the endogenous production of tumor necrosis factor alpha required as a costimulatory factor for interferon gamma-induced activation. Proc Natl. Acad. Sci. U.S.A..

[b0185] Powrie F., Menon S., Coffman R.L. (1993). Interleukin-4 and interleukin-10 synergize to inhibit cell-mediated immunity in vivo. Eur. J. Immunol..

[b0190] Qi L.S., Larson M.H., Gilbert L.A., Doudna J.A., Weissman J.S., Arkin A.P., Lim W.A. (2013). Repurposing CRISPR as an RNA-guided platform for sequence-specific control of gene expression. Cell.

[b0195] Quesniaux V.J., Nicolle D.M., Torres D., Kremer L., Guérardel Y., Nigou J., Puzo G., Erard F., Ryffel B. (2004). Toll-like receptor 2 (TLR2)-dependent-positive and TLR2-independent-negative regulation of proinflammatory cytokines by mycobacterial lipomannans. J. Immunol..

[b0200] Ran F.A., Hsu P.D., Wright J., Agarwala V., Scott D.A., Zhang F. (2013). Genome engineering using the CRISPR-Cas9 system. Nat. Protoc..

[b0205] Redford P.S., Murray P.J., O'Garra A. (2011). The role of IL-10 in immune regulation during M. tuberculosis infection. Mucosal Immunol..

[b0210] Reed J.M., Branigan P.J., Bamezai A. (2008). Interferon gamma enhances clonal expansion and survival of CD4+ T cells. J. Interferon Cytokine Res..

[b0215] Rock J.M., Hopkins F.F., Chavez A., Diallo M., Chase M.R., Gerrick E.R., Pritchard J.R., Church G.M., Rubin E.J., Sassetti C.M., Schnappinger D., Fortune S.M. (2017). Programmable transcriptional repression in mycobacteria using an orthogonal CRISPR interference platform. Nature Microbiology.

[b0220] Rose S.J., Babrak L.M., Bermudez L.E. (2015). Mycobacterium avium Possesses Extracellular DNA that Contributes to Biofilm Formation, Structural Integrity, and Tolerance to Antibiotics. PLoS ONE.

[b0225] Sambandan D., Dao D.N., Weinrick B.C., Vilchèze C., Gurcha S.S., Ojha A., Kremer L., Besra G.S., Hatfull G.F., Jacobs W.R. (2013). Keto-mycolic acid-dependent pellicle formation confers tolerance to drug-sensitive Mycobacterium tuberculosis. mBio.

[b0230] Sassetti C.M., Boyd D.H., Rubin E.J. (2003). Genes required for mycobacterial growth defined by high density mutagenesis. Mol. Microbiol..

[b0235] Schnaar R.L. (2015). Glycans and glycan-binding proteins in immune regulation: A concise introduction to glycobiology for the allergist. J. Allergy Clin. Immunol..

[b0240] Schroder K., Hertzog P.J., Ravasi T., Hume D.A. (2004). Interferon-gamma: an overview of signals, mechanisms and functions. J. Leukoc. Biol..

[b0245] Shalaby M.R., Waage A., Espevik T. (1989). Cytokine regulation of interleukin 6 production by human endothelial cells. Cell Immunol..

[b0250] Singh A.K., Carette X., Potluri L.P., Sharp J.D., Xu R., Prisic S., Husson R.N. (2016). Investigating essential gene function in Mycobacterium tuberculosis using an efficient CRISPR interference system. Nucleic Acids Res..

[b0255] Tebruegge M., Dutta B., Donath S., Ritz N., Forbes B., Camacho-Badilla K., Clifford V., Zufferey C., Robins-Browne R., Hanekom W., Graham S.M., Connell T., Curtis N. (2015). Mycobacteria-Specific Cytokine Responses Detect Tuberculosis Infection and Distinguish Latent from Active Tuberculosis. Am. J. Respir. Crit. Care Med..

[b0260] Vieira P., de Waal-Malefyt R., Dang M.N., Johnson K.E., Kastelein R., Fiorentino D.F., deVries J.E., Roncarolo M.G., Mosmann T.R., Moore K.W. (1991). Isolation and expression of human cytokine synthesis inhibitory factor cDNA clones: homology to Epstein- Barr virus open reading frame BCRFI. Proc. Natl. Acad. Sci. U.S.A..

[b0265] Vignal C., Guérardel Y., Kremer L., Masson M., Legrand D., Mazurier J., Elass E. (2003). Lipomannans, but not lipoarabinomannans, purified from Mycobacterium chelonae and Mycobacterium kansasii induce TNF-alpha and IL-8 secretion by a CD14-toll-like receptor 2-dependent mechanism. J. Immunol..

[b0270] Wajant H., Pfizenmaier K., Scheurich P. (2003). Tumor necrosis factor signaling. Cell Death Differ..

[b0275] World Health Organization. (2020, October 15). “Global tuberculosis report 2020” Retrieved from https://www.who.int/publications/i/item/9789240013131.

[b0280] Yuan C., Qu Z.L., Tang X.L., Liu Q., Luo W., Huang C., Pan Q., Zhang X.L. (2019). Mycobacterium tuberculosis Mannose-Capped Lipoarabinomannan Induces IL-10-Producing B Cells and Hinders CD4+Th1 Immunity. iScience.

